# Impact of *Plantago ovata* Forsk leaf extract on morpho-physio-biochemical attributes, ions uptake and drought resistance of wheat (*Triticum aestivum* L.) seedlings

**DOI:** 10.3389/fpls.2022.999170

**Published:** 2022-09-20

**Authors:** Khadiga Alharbi, Haifa Abdulaziz Sakit Alhaithloul, Aisha A. M. Alayafi, Wafa’a A. Al-Taisan, Suliman Mohammed Alghanem, Amina A. M. Al-Mushhin, Mona H. Soliman, Moodi Saham Alsubeie, Dan C. Vodnar, Romina Alina Marc

**Affiliations:** ^1^Department of Biology, College of Science, Princess Nourah bint Abdulrahman University, Riyadh, Saudi Arabia; ^2^Biology Department, College of Science, Jouf University, Sakaka, Saudi Arabia; ^3^Biological Sciences Department, Faculty of Science, University of Jeddah, Jeddah, Saudi Arabia; ^4^Department of Biology, College of Science, Imam Abdulrahman Bin Fasial University, Dammam, Saudi Arabia; ^5^Department of Biology, Faculty of Science, Tabuk University, Tabuk, Saudi Arabia; ^6^Department of Biology, College of Science and Humanities in Al-Kharj, Prince Sattam Bin Abdulaziz University, Al-Kharj, Saudi Arabia; ^7^Department of Botany and Microbiology, Faculty of Science, Cairo University, Giza, Egypt; ^8^Department of Biology, Faculty of Science, Taibah University, Yanbu, Saudi Arabia; ^9^Department of Biology, College of Science, Imam Mohammad Ibn Saud Islamic University (IMSIU), Riyadh, Saudi Arabia; ^10^Institute of Life Sciences, Faculty of Food Science and Technology, University of Agricultural Sciences and Veterinary Medicine, Cluj-Napoca, Romania; ^11^Department of Food Engineering, Faculty of Food Science and Technology, University of Agricultural Science and Veterinary Medicine Cluj-Napoca, Cluj-Napoca, Romania

**Keywords:** agronomic crop, antioxidant compounds, isabgol extract, nutrient uptake, oxidative stress, water deficit stress

## Abstract

The present study was conducted to examine the potential role of *Plantago ovata* Forsk leaf extract (POLE) which was applied at various concentration levels (control, hydropriming, 10, 20, 30, and 40% POLE) to the wheat (*Triticum aestivum* L.) seedlings. Drought stressed was applied at 60% osmotic potential (OM) to the *T. aestivum* seedlings to study various parameters such as growth and biomass, photosynthetic pigments and gas exchange characteristics, oxidative stress and response of various antioxidants and nutritional status of the plants. Various growth parameters such as gaseous exchange attributes, antioxidants and nutritional status of *T. aestivum* were investigated in this study. It was evident that drought-stressed condition had induced a negative impact on plant growth, photosynthetic pigment, gaseous exchange attributes, stomatal properties, and ion uptake by different organs (roots and shoots) of *T. aestivum*. The decrease in plant growth resulted from oxidative stress and overcome by the antioxidant (enzymatic and non-enzymatic) compounds, since their concentration increased in response to dehydration. Seed priming with POLE positively increased plant growth and photosynthesis, by decreasing oxidative stress indicators and increasing activities of antioxidant (enzymatic and non-enzymatic) compounds, compared to the plants which were grown without the application of POLE. Our results also depicted that optimum concentration of POLE for *T. aestivum* seedlings under drought condition was 20%, while further increase in POLE (30 and 40%) induced a non-significant (*P* < 0.05) effect on growth (shoot and root length) and biomass (fresh and dry weight) of *T. aestivum* seedling. Here we concluded that the understanding of the role of seed priming with POLE in the increment of growth profile, photosynthetic measurements and nutritional status introduces new possibilities for their effective use in drought-stressed condition and provides a promising strategy for *T. aestivum* tolerance against drought-stressed condition.

## Introduction

Clean sufficient water supplies are vital for all communities, industries, and ecosystems for drinking, farming, sanitation, and energy production. Yet, the world’s water systems face formidable threats. UNESCO (World Water Assessment Programme, 2012) estimates that around 700 million people in 43 countries suffer from water scarcity and predict that “by 2025, 1.8 billion people will be living in countries or regions with absolute water scarcity, and two-thirds of the world’s population could be living under water stressed conditions.” Pakistan is already experiencing a shortage in freshwater resources. In the wake of growing population, per capita water availability declined from 5,600 m^3^ in 1951 to 1,200 m^3^ in 2003, and is reaching close to the water scarcity threshold level of 1,000 m^3^ (Waraich et al., 2011; Aslam et al., 2013). A proper amount of soil moisture is compulsory for crop growth, transpiration, and also for transportation of food prepared in leaves to sink and in drought condition, crop growth, and yield decreases (Hasanuzzaman et al., 2018; Rana et al., 2020; Saleem et al., 2020b,d). Drought conditions can lead to a number of changes in plants, including reduced growth, lower fresh and dry biomass, lower rates of photosynthesis, and reduced absorption of essential nutrients from the soil (Ahmad et al., 2017; Akram et al., 2018; Khan et al., 2019a; Dola et al., 2022; Farooq et al., 2022; Faryal et al., 2022). Reductions in chlorophyll content and oxidation of membrane lipids and proteins are the crucial reasons for plant damage caused by the production of drought-induced highly reactive oxygen species (ROS), leading to the modification of the cellular redox status (Alam et al., 2020; Saleem et al., 2020c, 2022; Yaseen et al., 2020; Afridi et al., 2022; Wahab et al., 2022). However, the negative impacts of drought depend on the total length of the plant growth cycle, the degree of drought stress and frequency of drought conditions, growth conditions, and plant species (Wu et al., 2018; Goharrizi et al., 2020; Sakya and Prahasto, 2020). Under environmental stress, imbalance in ROS accumulation and generation can occur, which results in the formation of hydrogen peroxide (H_2_O_2_), superoxide radicles (O^–2^), and hydroxide ions (OH), all of which are known as stress indicators at cellular levels (Imran et al., 2020a,b; Ma et al., 2022a, b). These ROS are toxic to plants and scavenged by various types of antioxidants like SOD (superoxide dismutase), POD (peroxidase), catalase (CAT), and ascorbate peroxidase (APX), etc. to maintain cellular homeostasis (Imran et al., 2019; Rehman et al., 2019; Saleem et al., 2020a,e,g). Previously, many researchers concluded that varieties of antioxidants have increased their activities under drought environments in *Brassica napus* L. (Khan et al., 2019b), *Triticum aestivum* (Nikolaeva et al., 2010), and *Zea mays* L. (Anjum et al., 2017). Additionally, drought conditions reduce the photosynthetic rates, expansion of leaves, increase stomatal closure, the levels of ROS and early leaf catabiotic, and decrease the translocation within the plant, resulting in overall decreased crop yield (Ahmad et al., 2017; Anjum et al., 2017). Adaptive agricultural strategies are urgently needed in these changing environments (Singh et al., 2022). Because different organs show various levels of sensitivity to water stress, a whole-plant approach is required in research rather than focusing only on single components (Liang et al., 2018).

Wheat (*Triticum aestivum* L.) is grown in nearly all parts of the world (Ahmad et al., 2022; Khan et al., 2023). Pakistan stands fourth in Asia and eleventh in the world as far as wheat production are concerned (Mahmood et al., 2020; Saeed et al., 2022) and in Pakistan, the worst threat for the production of wheat is drought (Ahmad et al., 2017). In addition, wheat is a main grain cereal widely consumed worldwide and is a staple food for more than 50% of world population (Azooz et al., 2012; Amna Ali et al., 2021; Adnan et al., 2022). Physiological responses in wheat include closure of stomata, decrease in the activity of photosynthesis, development of oxidative stress, alteration in the integrity of cell wall, production of metabolites which are toxic and cause plants’ death, signal recognition of roots, turgor loss and adjustment of osmosis, reduction in water potential of leaf, decrease in stomata conductance to CO_2_, reduction of internal CO_2_ concentration, and reduction of growth rates (Nezhadahmadi et al., 2013; Mehmood et al., 2021; Zainab et al., 2021; Saleem et al., 2022). According to researchers, there is a relationship between different physiological responses of wheat and their resistance functions under drought such as high amount of relative water and potential water (Dimkpa et al., 2020; Yaseen et al., 2020). Cysteine is expressed in wheat leaf organs and its contribution in proteolysis activity rises under drought (Dehghani and Mostajeran, 2020). According to the study of Denčić et al. (2000), wheat is paid special attention due to its morphological traits during drought stress including leaf (shape, expansion, area, size, senescence, pubescence, waxiness, and cuticle tolerance) and root (dry weight, density, and length). Although due to the shortage of large genetic tolerant assets and the intricacy in physiological, biochemical and genetic attributes, the progress toward water resistance cultivars is considerably vulnerable. Therefore, identification of numerous genetic ways and also to recognize the strategies of water stress tolerance that might lead to high levels of drought resistance is more important.

Different approaches are being explored to resolve this issue and to improve the crop yields (Zaheer et al., 2020a,b). These approaches include the development of stress-tolerant genotypes (Singh et al., 2022), use of plant extract, or enhance stress tolerance by the exogenous applications of various organic compounds (Haider et al., 2022). The response of plants to water stress depends on the growth stage, duration, and species. The most sensitive stage of the plant life cycle is the reproductive stage that significantly influences the final productivity (Fahad et al., 2017). Accordingly the increase in the use of plant extracts depends or seed priming with various chemicals depends up on the concentration of the substance used and growth stage of plant. Seed priming is known as pre-sowing treatment that involves the exposure of seeds to lower external water potential (Khan et al., 2019b). Recent evidences showed that seed priming improves stress-tolerance throughout the subsequent plant growth and development stages. Although the germination process is not obvious during the process of seed priming, it is advised that priming initiates metabolic accomplishments required for embryonic growth and radical projection (Farooq et al., 2013; Hassini et al., 2017). As a consequence of priming, germination rates increase than emergence of uniform seedling compared with non-primed seeds (Jiang et al., 2016). Recently, *Plantago ovata* (also known as desert Indian wheat or ispaghula) leaf extract becomes interestingly popular in ameliorating abiotic stress in many crops (Asgharipour and Rafiei, 2010; Rahimi et al., 2010). *Plantago ovata* is an important medicinal plant which has different compounds such as phenolic compounds (caffeic acid derivatives), flavonoids, alkaloids, terpenoids, vitamin C, antioxidants, and anti-inflammatory agents (Saghir et al., 2008). A hypothesis made that POLE extract as an agent against abiotic stress may be studied in wheat as a drought-tolerant agent. Hence, we conducted the current study to determine (i) the impact of drought stress on seed germination, plant growth and biomass, photosynthetic pigment, gaseous exchange attributes, stomatal properties and nutritional status in wheat, when primed with *P. ovata* extract and (ii) various oxidative stress indicators and the response of antioxidant (enzymatic and non-enzymatic) compounds in the roots and leaves of wheat seedlings. According to best of our knowledge, this study is among the few studies which focus on the drought tolerance and resistance using POLE in wheat in order to investigate their suitability for water-deficient conditions. Findings from the present study will add to our understanding the mechanism of drought tolerance and resistance using POLE in *T. aestivum* seedlings.

## Materials and methods

### Experimental design and growth conditions

‘V12304’ wheat line was used for this experiment and this line is more tolerant to drought stress than other wheat lines and has been used in a number of previous studies conducted under drought stress conditions (Zulkiffal et al., 2018). Seeds were placed in Petri dishes, which were placed in growth chamber (100 W, Guangdong PHILIPS Co., Guangdong, China) with a day/night temperature of 25 ± 2°C and day/night humidity of 80%. Before starting a Petri dish experiment, the seeds were surface-sterilized with 0.1% HgCl_2_ for the prevention of surface fungal/bacterial contamination, followed by de-ionized water and for priming seeds that were soaked in the POLE solutions of varying concentrations (control, hydropriming, 10, 20, 30, and 40% POLE). *Plantago ovata* leaf was collected from the garden and the leaves of *P. ovata* were washed carefully with distilled water and then dried and crushed with the help of grinding machine. Then, the filtrate (as it is water soluble) was used for seed priming at various concentrations. After 12 h of seeds soaked in the various levels of POLE, the seeds were dried to attain their original moisture contents with the help of blotting papers. The effect of control and drought stressed environment in wheat grown under the seed priming with POLE is shown in Supplementary Figure 1. The Petri dishes used in this study for wheat lines have up and down (two) filter papers. Three replicates with 15 seedlings were used for each treatment and were arranged in completely randomized design (CRD). All seedlings were also divided into two subgroups: (I) control [100% OM (osmotic potential)] and (II) drought [60% OM [using polyethylene glycol (PEG-8000)]. The plant seedlings were subjected to PEG-8000 concentration along with control treatments for 24 h. About 10 mL of Hoagland’s (compositions are given in Supplementary Table 1) and PEG solution were given to each Petri dish, and water levels and other intercultural operations were monitored on daily basis. All seedlings were harvested after 21 days, from the beginning of the experiment for measuring different traits. All seedlings were harvested for measuring various morphological, physiological, and nutritional status from wheat seedlings.

### Morphological traits and data collection

All seedlings were rooted-up in July 2019 to study different growth, germination, and other morphological and physiological parameters. Analysis of different biological parameters was performed in College of Agriculture and Food Sciences, King Faisal University, Al-Ahsa 31982, Saudi Arabia. The leaf in each treatment was picked at a rapid growth stage during 09:00–10:30 a.m. The sampled leaves were washed with distilled water, immediately placed in liquid nitrogen, and stored in a freezer at low temperature (−80°C) for further analysis. Germination index, time to 50% germination, co-efficient of uniformity of emergence, mean germination time, and germination energy (E) were measured by following the method presented by Coolbear et al. (1984), Wiesner (1990), Ruan et al. (2002), and Bewley and Black (2013). Germination percentage was calculated by the following formula:

G%=No.of⁢germinated⁢seeds/total⁢number⁢of⁢seeds× 100

Stomata were counted at random in 30 visual sections on the abaxial epidermis, and final tallies were used to calculate stomatal density. We used Image J software (Schneider et al., 2012) for measuring stomatal lengths, widths, and apertures.

Plants in each treatment were harvested and separated into roots and shoots for growth and morphology traits. Shoot length was defined as the length of the plant from the surface growth medium line of the Petri dish to the tip of the uppermost shoot and root length was also measured. Shoot fresh weight was measured by measuring the weight of shoots with the help of a digital weighing balance and root fresh weight was also measured. After that plant samples were oven-dried for 1 h at 105°C, then 65°C for 72 h until the weight become uniform, and dry biomass was recorded. Roots were washed with distilled water and dipped in 20 mM Na_2_EDTA for 15–20 min, washed thrice with distilled water and finally with de-ionized water, and then oven-dried for further analysis.

### Determination of photosynthetic pigments and gas exchange parameters

For chlorophyll determination, 0.1 g of fresh leaf sample was extracted with 8 mL of 95% acetone for 24 h at 4°C in the dark. The absorbance was measured by a spectrophotometer (UV-2550; Shimadzu, Kyoto, Japan) at 646.6, 663.6, and 450 nm. Chlorophyll content was calculated by the standard method of Arnon (1949) and Ali et al. (2022a,c).

Gas exchange parameters were also measured during the same day. Net photosynthesis (*Pn*), leaf stomatal conductance (*Gs*), transpiration rate (*Ts*), and intercellular carbon dioxide concentration (*Ci*) were measured from three different plants in each treatment group. Measurements were conducted between 11:30 and 13:30 on days with clear sky. Rates of leaf *Pn*, *Gs, Ts*, and *Ci* were measured with a LI-COR gas-exchange system (LI-6400; LI-COR Biosciences, Lincoln, NE, United States) with a red-blue LED light source on the leaf chamber. In the LI-COR cuvette, CO_2_ concentration was set as 380 mmol mol^–1^ and LED light intensity was set at 1,000 mmol m^–2^ s^–1^, which is the average saturation intensity for photosynthesis in *T. aestivum* (Austin, 1990).

### Determination of oxidative stress indicators

The degree of lipid peroxidation was evaluated as malondialdehyde (MDA) contents. Briefly, 0.1 g of frozen leaves were ground at 4°C in a mortar with 25 mL of 50 mM phosphate buffer solution (pH 7.8) containing 1% polyethene pyrrole. The homogenate was centrifuged at 10,000 × *g* at 4°C for 15 min. The mixtures were heated at 100°C for 15–30 min and then quickly cooled in an ice bath. The absorbance of the supernatant was recorded using a spectrophotometer (xMark™ Microplate Absorbance Spectrophotometer; Bio-Rad, United States) at wavelengths of 532, 600, and 450 nm. Lipid peroxidation was expressed as l mol g^–1^ using the formula: 6.45 (A532-A600)-0.56 A450. Lipid peroxidation was measured using a method previously published by Heath and Packer (1968) and Nawaz et al. (2022).

To estimate H_2_O_2_ content of plant tissues (root and leaf), 3 mL of sample extract was mixed with 1 mL of 0.1% titanium sulfate in 20% (v/v) H_2_SO_4_ and centrifuged at 6,000 × g for 15 min. The yellow color intensity was evaluated at 410 nm. The H_2_O_2_ level was computed by extinction coefficient of 0.28 mmol^–1^ cm^–1^. The contents of H_2_O_2_ were measured by the method presented by Jana and Choudhuri (1981).

Stress-induced electrolyte leakage (EL) of uppermost stretched leaves was determined using methodology of Dionisio-Sese and Tobita (1998) and Ali et al. (2022d). The leaves were cut into minor slices (5 mm length) and placed in test tubes having 8-mL distilled water. These tubes were incubated and transferred into water bath for 2 h prior to measuring the initial electrical conductivity (EC_1_). The samples were autoclaved at 121°C for 20 min, and then cooled down to 25°C before measuring the final electrical conductivity (EC_2_). Electrolyte leakage was calculated using the following formula:

$$\text{EL}=\left({\text{EC}}_{1}/{\text{EC}}_{2}\right)\times 100$$

### Determination of antioxidant enzyme activities

To evaluate enzyme activities, fresh leaves (0.5 g) were homogenized in liquid nitrogen and 5 mL of 50 mmol sodium phosphate buffer (pH 7.0) including 0.5 mmol EDTA and 0.15 mol NaCl. The homogenate was centrifuged at 12,000 × *g* for 10 min at 4°C, and the supernatant was used for the measurement of SOD and POD activities. Superoxidase dismutase activity was assayed in 3-mL reaction mixture containing 50 mM sodium phosphate buffer (pH 7), 56 mM nitro blue tetrazolium, 1.17 mM riboflavin, 10 mM methionine, and 100 μL enzyme extract. Finally, the sample was measured using a spectrophotometer (xMark™ Microplate Absorbance Spectrophotometer; Bio-Rad). Enzyme activity was measured using a method by Chen and Pan (1996) and expressed as U g^–1^ FW.

Peroxidase activity in the leaves was estimated using the method of Sakharov and Ardila (1999) and Ali et al. (2022b) using guaiacol as the substrate. A reaction mixture (3 mL) containing 0.05 mL of enzyme extract, 2.75 mL of 50 mM phosphate buffer (pH 7.0), 0.1 mL of 1% H_2_O_2_, and 0.1 mL of 4% guaiacol solution was prepared. Increases in the absorbance at 470 nm because of guaiacol oxidation were recorded for 2 min. One unit of enzyme activity was defined as the amount of the enzyme.

Catalase activity was analyzed according to Aebi (1984). The assay mixture (3.0 mL) was comprised of 100 μL enzyme extract, 100 μL H_2_O_2_ (300 mM), and 2.8 mL 50 mM phosphate buffer with 2 mM ETDA (pH 7.0). The CAT activity was measured from the decline in absorbance at 240 nm as a result of H_2_O_2_ loss (ε = 39.4 mM^–1^ cm^–1^).

Ascorbate peroxidase activity was measured according to Nakano and Asada (1981). The mixture containing 100 μL enzyme extract, 100 μL ascorbate (7.5 mM), 100 μL H_2_O_2_ (300 mM), and 2.7 mL 25 mM potassium phosphate buffer with 2 mM EDTA (pH 7.0) was used for measuring APX activity. The oxidation pattern of ascorbate was estimated from the variations in wavelength at 290 nm (ε = 2.8 mM^–1^ cm^–1^).

### Determination of non-enzymatic antioxidants, sugars, and proline contents

Plant ethanol extracts were prepared for the determination of non-enzymatic antioxidants and some key osmolytes. For this purpose, 50 mg of plant dry material was homogenized with 10 mL ethanol (80%) and filtered through Whatman No. 41 filter paper. The residue was re-extracted with ethanol and the two extracts were pooled together to a final volume of 20 mL. The determination of flavonoids (Pêkal and Pyrzynska, 2014), phenolics (Bray and Thorpe, 1954), ascorbic acid (Azuma et al., 1999), anthocyanin (Lewis et al., 1998), and total sugars (Dubois et al., 1956) was performed from the extracts.

Fresh leaf material (0.1 g) was mixed thoroughly in 5-mL aqueous sulpho salicylic acid (3%). The mixture was centrifuged at 10,000 × g for 15 min and aliquot (1 mL) was poured into a test tube having 1 mL acidic ninhydrin and 1 mL glacial acetic acid. The reaction mixture was first heated at 100°C for 10 min and then cooled in an ice bath. The reaction mixture was extracted with 4-mL toluene and test tubes are vortexed for 20 s and cooled. Thereafter, the light absorbance at 520 nm was measured by using UV–VIS spectrophotometer (Hitachi U-2910, Tokyo, Japan). The free proline content was determined on the basis of standard curve at 520 nm absorbance and expressed as μmol (g FW) ^–1^ (Bates et al., 1973).

### Determination of nutrient contents

For nutrient analysis, plant roots and shoots were washed twice in redistilled water, dipped in 20 mM EDTA for 3 s and then, again washed with deionized water twice for the removal of adsorbed metal on plant surface. The washed samples were then oven-dried for 24 h at 105°C. The dried roots and shoots were digested using wet digestion method in HNO_3_: HClO_4_ (7:3 V/V) until clear samples were obtained. Each sample was filtered and diluted with redistilled water up to 50 mL. The root and shoot contents of Fe^2+^, Mg^2+^, Ca^2+^, and P and were analyzed using Atomic Absorption Spectrophotometer (AAS) model Agilent 240FS-AA.

### Statistical analysis

Statistical analysis of data was performed with analysis of variance (ANOVA) using a statistical program Co-Stat version 6.2, Cohorts Software, 2003, Monterey, CA, United States. All the data obtained were tested by one-way ANOVA. Thus, the differences between treatments were determined using ANOVA, and the least significant difference test (*P* < 0.05) was used for multiple comparisons between treatment means. Logarithmic or inverse transformations were performed for data normalization, where necessary, prior to analysis. Pearson’s correlation analysis was performed to quantify relationships between various analyzed variables. The graphical presentation was carried out using Origin-Pro 2017. The RStudio (R Core Team, 2018) was used to calculate Pearson’s correlation through heat-map analysis. Furthermore, the plots of principal component analysis and heat map on *T. aestivum* parameters were carried out using the RStudio.

## Results

### Effect of different concentrations of *Plantago ovata* Forsk leaf extract on seed germination and growth of wheat grown under well-watered and drought-stressed condition

**FIGURE 1:**
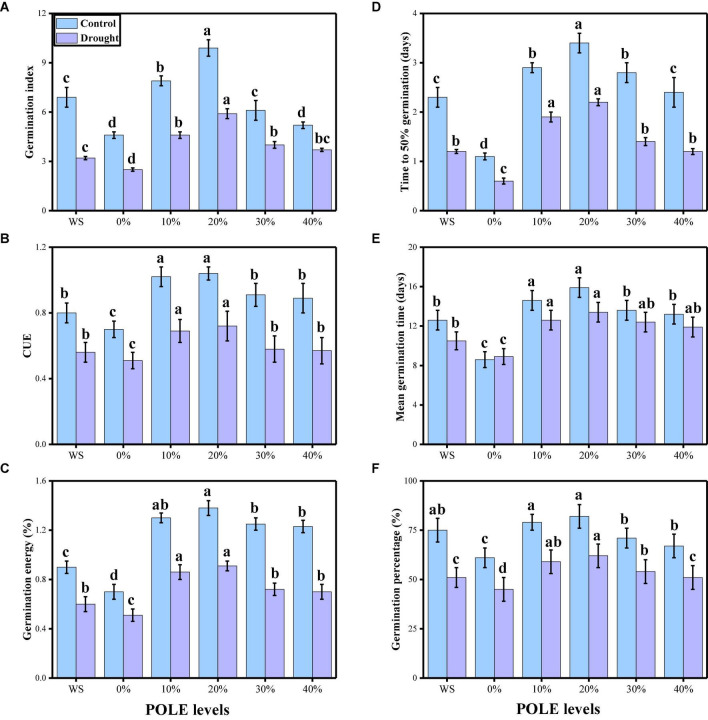
Effect of seed priming with *Plantago ovata* Forsk leaf extract (POLE) on germination index **(A)**, time to total 50% germination **(B)**, co-efficient of uniformity of emergence **(C)**, mean germination time **(D)**, germination energy **(E)**, and germination percentage **(F)** under well-watered and drought-stressed environment in wheat. Means sharing similar letter(s) within a column for each parameter do not differ significantly at *P* < 0.05. Data in the figures are means of four repeats (*n* = 4) of just one harvest of wheat plants ± standard deviation (SD). Different lowercase letters on the error bars indicate significant difference between the treatments. Different treatments of POLE used in this study are as follows: WS (Control, without POLE + without water), 0% (0% POLE), 10% (10% POLE), 20% (20% POLE), 30% (30% POLE), and 40% (40% POLE).

**FIGURE 2:**
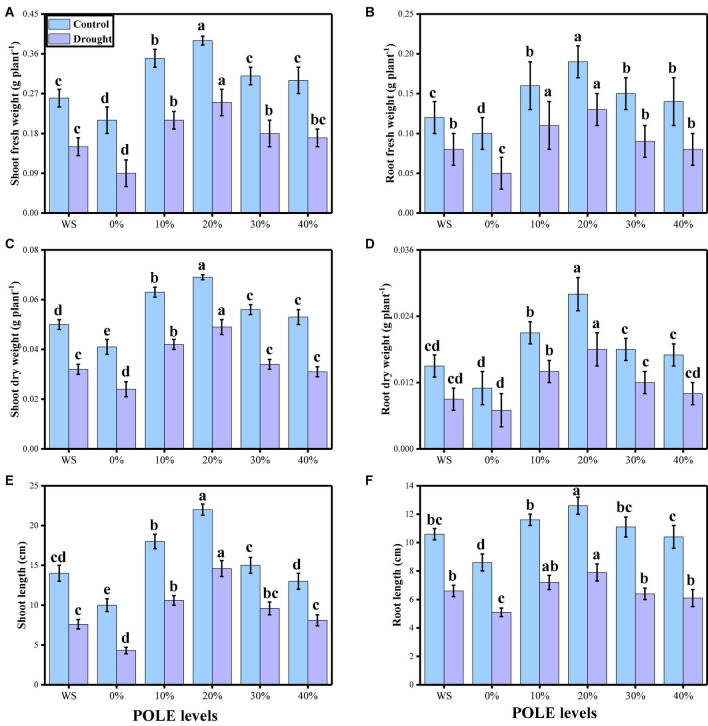
Effect of seed priming with *Plantago ovata* Forsk leaf extract (POLE) on shoot fresh weight **(A)**, root fresh weight **(B)**, shoot dry weight **(C)**, root dry weight **(D)**, shoot length **(E)**, and root length **(F)** under well-watered and drought-stressed environment in wheat. Means sharing similar letter(s) within a column for each parameter do not differ significantly at *P* < 0.05. Data in the figures are means of four repeats (*n* = 4) of just one harvest of wheat plants ± standard deviation (SD). Different lowercase letters on the error bars indicate significant difference between the treatments. Different treatments of POLE used in this study are as follows: WS (Control, without POLE + without water), 0% (0% POLE), 10% (10% POLE), 20% (20% POLE), 30% (30% POLE), and 40% (40% POLE).

In the present study, the effects of different concentrations of POLE (control, hydropriming, 10, 20, 30, and 40% POLE) on seed germination, plant growth, and biomass were also determined in wheat seedlings when grown in well-watered and drought-stressed environment. The data regarding various germination parameters are presented in Figure 1, and data regarding various growth and morphological parameters of wheat seedlings are presented in Figure 2. The results showed that drought-stressed condition significantly (*P* < 0.05) increased germination index, time to 50% germination, co-efficient of uniformity of emergence, mean germination time, germination energy (E), germination percentage, shoot fresh weight, root fresh weight, shoot dry weight, root dry weight, shoot length, and root length in wheat seedlings, compared to those plants which were grown in well-watered environment. In addition, it was depicted that seed priming with POLE significantly (*P* < 0.05) increased germination index, time to 50% germination, co-efficient of uniformity of emergence, mean germination time, germination energy (E), germination percentage, shoot fresh weight, root fresh weight, shoot dry weight, root dry weight, shoot length, and root length in well-watered and drought-stressed plants, when compared with plant grown without POLE (0% POLE or hydropriming). Seed priming with POLE showing more significant results up to a level of 20% POLE, while further increase in the POLE levels (30 and 40% POLE) induces a negative impact of all seed germination parameters and growth in wheat seedlings in well-watered and drought-stressed environment.

### Effect of different concentrations of *Plantago ovata* Forsk leaf extract on gaseous exchange parameters and stomatal properties of wheat grown under well-watered and drought-stressed condition

**FIGURE 3:**
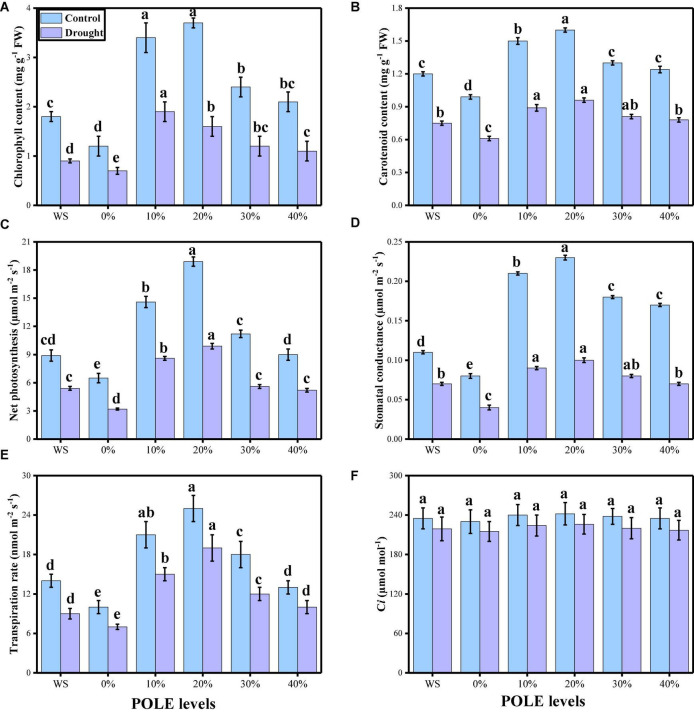
Effect of seed priming with *Plantago ovata* Forsk leaf extract (POLE) on total chlorophyll contents **(A)**, carotenoid contents **(B)**, net photosynthesis **(C)** stomatal conductance **(D)**, transpiration rate **(E)**, and intercellular CO_2_ **(F)** under well-watered and drought-stressed environment in wheat. Means sharing similar letter(s) within a column for each parameter do not differ significantly at *P* < 0.05. Data in the figures are means of four repeats (*n* = 4) of just one harvest of wheat plants ± standard deviation (SD). Different lowercase letters on the error bars indicate significant difference between the treatments. Different treatments of POLE used in this study are as follows: WS (Control, without POLE + without water), 0% (0% POLE), 10% (10% POLE), 20% (20% POLE), 30% (30% POLE), and 40% (40% POLE).

**FIGURE 4:**
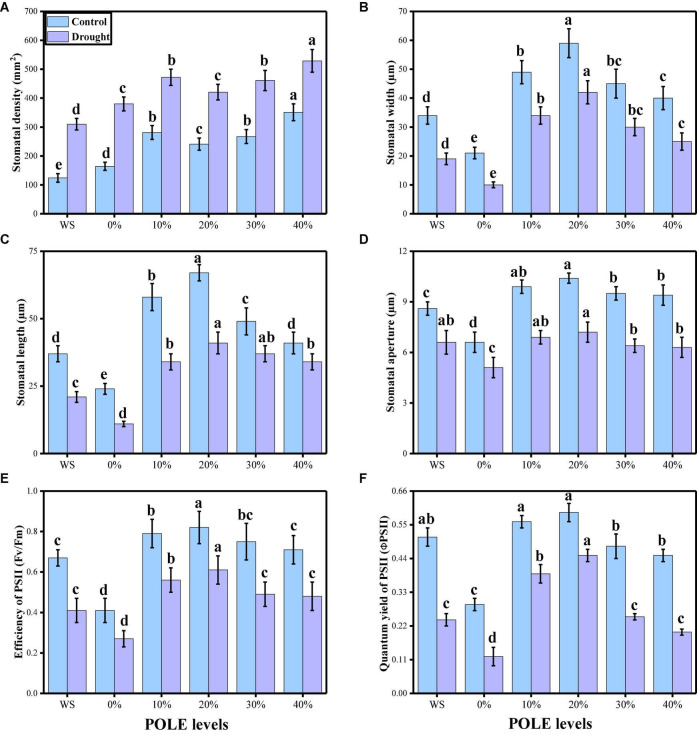
Effect of seed priming with *Plantago ovata* Forsk leaf extract (POLE) on stomatal density **(A)**, stomatal width **(B)**, stomatal length **(C)**, stomatal aperture **(D)**, efficiency of PSII **(E)**, and quantum yield of PSII **(F)** under well-watered and drought stressed environment in wheat. Means sharing similar letter(s) within a column for each parameter do not differ significantly at *P* < 0.05. Data in the figures are means of four repeats (*n* = 4) of just one harvest of wheat plants ± standard deviation (SD). Different lowercase letters on the error bars indicate significant difference between the treatments. Different treatments of POLE used in this study are as follows: WS (Control, without POLE + without water), 0% (0% POLE), 10% (10% POLE), 20% (20% POLE), 30% (30% POLE), and 40% (40% POLE).

Various photosynthetic pigments (chlorophyll and carotenoid contents), gas exchange parameters [net photosynthesis (*Pn*), leaf stomatal conductance (*Gs*), transpiration rate (*Ts*), and intercellular carbon dioxide concentration (*Ci*)], and stomatal properties (stomatal density, stomatal width, stomatal length, stomatal aperture, efficiency of PSII, and quantum yield of PSII) were also measured in this study. The data regarding photosynthetic pigments and gaseous exchange parameters are presented in Figure 3, and the data regarding the stomatal properties in wheat seedlings are presented in Figure 4 under the application of POLE in well-watered and drought-stressed environment. The results showing that the chlorophyll contents, carotenoid contents, net photosynthesis, leaf stomatal conductance, transpiration rate, stomatal width, stomatal length, stomatal aperture, efficiency of PSII, and quantum yield of PSII were decreased in the plant which were grown in drought stress condition (60% OM), when compared to the plants which were grown in the well-watered condition (100% OM). Although stomatal density was increased in the plants which were grown in drought stress environment and intercellular carbondioxide concentration showed non-significant increase or decrease in all treatments of drought stress and POLE. Results also showing that photosynthetic pigments, gas exchange parameters, and stomatal properties in drought-stressed condition can be increased with the seed priming with POLE which significantly (*P* < 0.05) increased chlorophyll contents, carotenoid contents, net photosynthesis, leaf stomatal conductance, transpiration rate, stomatal width, stomatal length, stomatal aperture, efficiency of PSII, and quantum yield of PSII, compared to those plants which were grown in 0% POLE or hydropriming. In addition, the maximum contents of photosynthetic pigments, gas exchange attributes and stomatal properties showed maximum results up to 20% POLE, while further increase in the concentration of POLE (30 and 40%) induced a significant (*P* < 0.05) decrease in all these parameters, compared to those plants which were grown in 0% POLE or hydropriming.

### Effect of different concentrations of *Plantago ovata* Forsk leaf extract on oxidative stress indicators and antioxidants response of wheat grown under well-watered and drought-stressed condition

**FIGURE 5:**
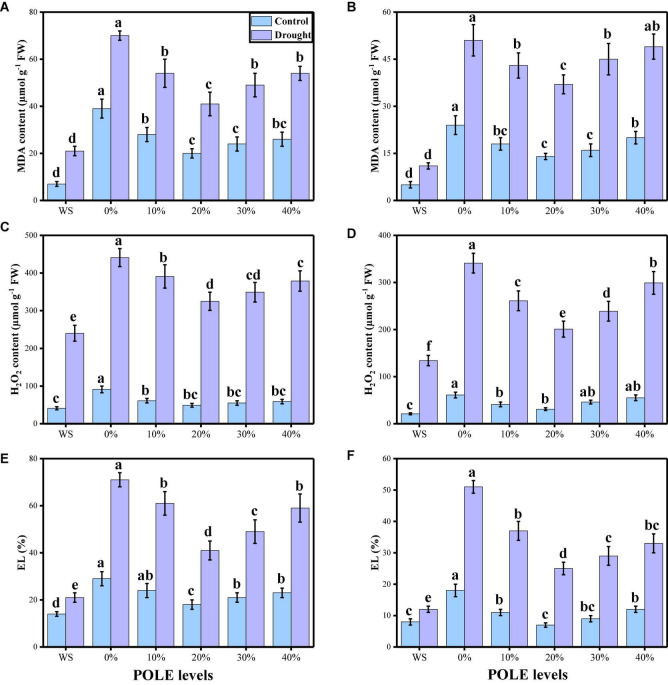
Effect of seed priming with *Plantago ovata* Forsk leaf extract (POLE) on MDA contents in the roots **(A)**, MDA contents in the leaves **(B)**, H_2_O_2_ contents in the roots **(C)**, H_2_O_2_ contents in the leaves **(D)**, EL percentage in the roots **(E)**, and EL percentage in the leaves **(F)** under well-watered and drought-stressed environment in wheat. Means sharing similar letter(s) within a column for each parameter do not differ significantly at *P* < 0.05. Data in the figures are means of four repeats (*n* = 4) of just one harvest of wheat plants ± standard deviation (SD). Different lowercase letters on the error bars indicate significant difference between the treatments. Different treatments of POLE used in this study are as follows: WS (Control, without POLE + without water), 0% (0% POLE), 10% (10% POLE), 20% (20% POLE), 30% (30% POLE), and 40% (40% POLE).

In the present study, we also elucidated different oxidative stress parameters (MDA, H_2_O_2_ initiation, and EL) in the roots and shoots of wheat seedlings. The data regarding different oxidative stress parameters are presented in Figure 5, under the application of POLE in well-watered and drought-stressed environment. According to the given results, the contents of MDA, H_2_O_2_ initiation and EL (%) were increased in the roots and shoots of the plant grown in drought stress (60% OM), when compared to the plants which were grown in the well-watered condition (100% OM). Seed priming with POLE decreased the contents MDA, H_2_O_2_ initiation, and EL (%) in the roots and shoots of the plant, when grown in well-watered and drought-stressed condition. Although the maximum decrease in the content MDA, H_2_O_2_ initiation and EL (%) was observed in 20% POLE, further increase in POLE (30 and 40%) increases contents MDA, H_2_O_2_ initiation, and EL (%) in the roots and shoots of the wheat seedlings in well-watered and drought stress condition, compared to the plants grown in 0% POLE.

**FIGURE 6:**
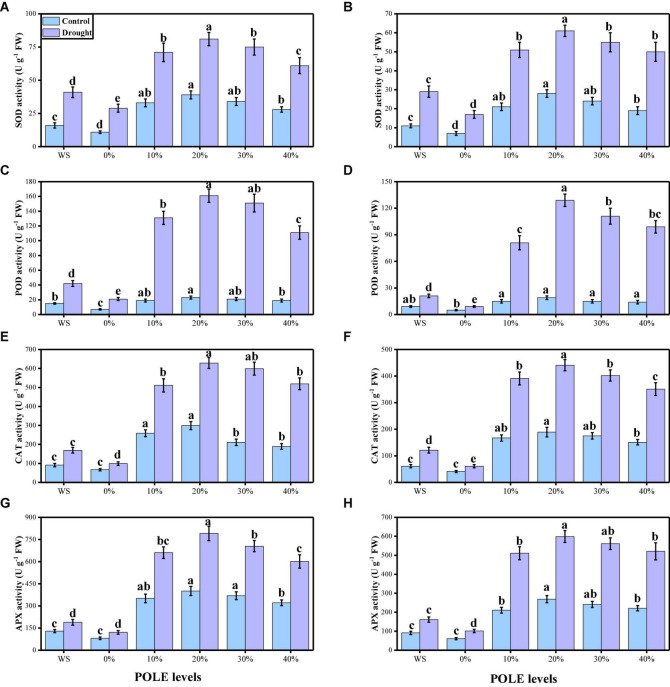
Effect of seed priming with *Plantago ovata* Forsk leaf extract (POLE) on SOD activity in the roots **(A)**, SOD activity in the leaves **(B)**, POD activity in the roots **(C)**, POD activity in the leaves **(D)**, CAT activity in the roots **(E)**, CAT activity in the leaves **(F)**, APX activity in the roots **(G)**, and APX activity in the leaves **(H)** under well-watered and drought-stressed environment in wheat. Means sharing similar letter(s) within a column for each parameter do not differ significantly at *P* < 0.05. Data in the figures are means of four repeats (*n* = 4) of just one harvest of wheat plants ± standard deviation (SD). Different lowercase letters on the error bars indicate significant difference between the treatments. Different treatments of POLE used in this study are as follows: WS (Control, without POLE + without water), 0% (0% POLE), 10% (10% POLE), 20% (20% POLE), 30% (30% POLE), and 40% (40% POLE).

**FIGURE 7:**
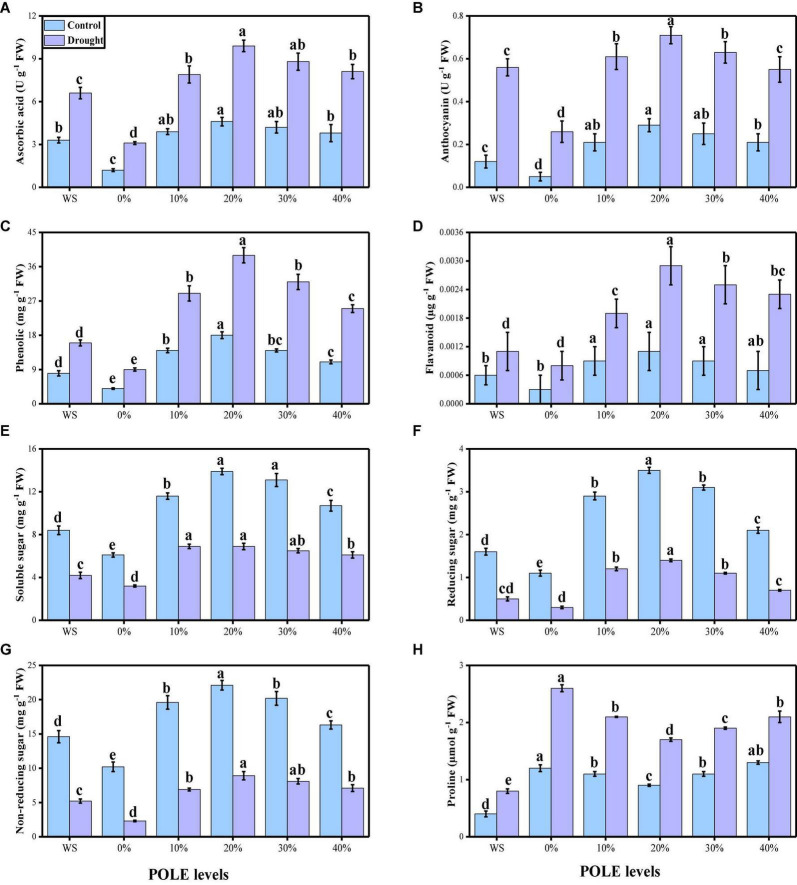
Effect of seed priming with *Plantago ovata* Forsk leaf extract (POLE) on ascorbic acid contents **(A)**, anthocyanin contents **(B)**, phenolic contents **(C)**, flavonoid contents **(D)**, soluble sugar contents **(E)**, reducing sugar contents **(F)**, non-reducing sugar contents **(G)**, and proline contents **(H)** under well-watered and drought-stressed environment in wheat. Means sharing similar letter(s) within a column for each parameter do not differ significantly at *P* < 0.05. Data in the figures are means of four repeats (*n* = 4) of just one harvest of wheat plants ± standard deviation (SD). Different lowercase letters on the error bars indicate significant difference between the treatments. Different treatments of POLE used in this study are as follows: WS (Control, without POLE + without water), 0% (0% POLE), 10% (10% POLE), 20% (20% POLE), 30% (30% POLE), and 40% (40% POLE).

The activities of various enzymatic (SOD, POD, CAT, and APX) and non-enzymatic (ascorbic acid, anthocyanin, phenolics, and flavonoids) antioxidants were also increased due to low water contents in the mixture. The data regarding enzymatic antioxidants are presented in Figure 6, and the data regarding non-enzymatic antioxidants in wheat seedlings are presented in Figure 7. Compared to the plants grown in the well-watered conditions, the activities of enzymatic antioxidants (SOD, POD, CAT, and APX) in the roots and leaves and compounds of non-enzymatic antioxidants (ascorbic acid, anthocyanin, phenolics, and flavonoids) of *T. aestivum* seedlings were increased significantly (*P* < 0.05) in the plants grown in the drought-stressed condition. The results also showing that the activities of enzymatic and non-enzymatic antioxidants were further increased with the increasing levels of POLE in well-watered and drought-stressed plants, compared with those plants of 0% POLE. Moreover, the maximum increased in the activities of enzymatic and non-enzymatic antioxidants were found in the plants which were grown in 20% POLE, while further increase such as 30 and 40% POLE induces a significant (*P* < 0.05) decrease in the activities of enzymatic and non-enzymatic antioxidants, compared with those plants which were not primed with POLE.

The contents of sugar (soluble, reducing, and non-reducing) and proline from the leaves of *T. aestivum* seedlings were also determined and presented in Figure 7. Compared to the well-watered, drought-stressed condition significantly (*P* < 0.05) decrease the contents of soluble, reducing and non-reducing sugar while increase the contents of proline in the leaves of wheat. Results show that the contents of soluble, reducing and non-reducing sugar were increased with the seed priming with POLE while they induce a significant decrease in the contents of proline, compared with those plants which were not primed with 0% POLE.

### Effect of different concentrations of *Plantago ovata* Forsk leaf extract on nutritional status of wheat grown under well-watered and drought-stressed condition

**FIGURE 8:**
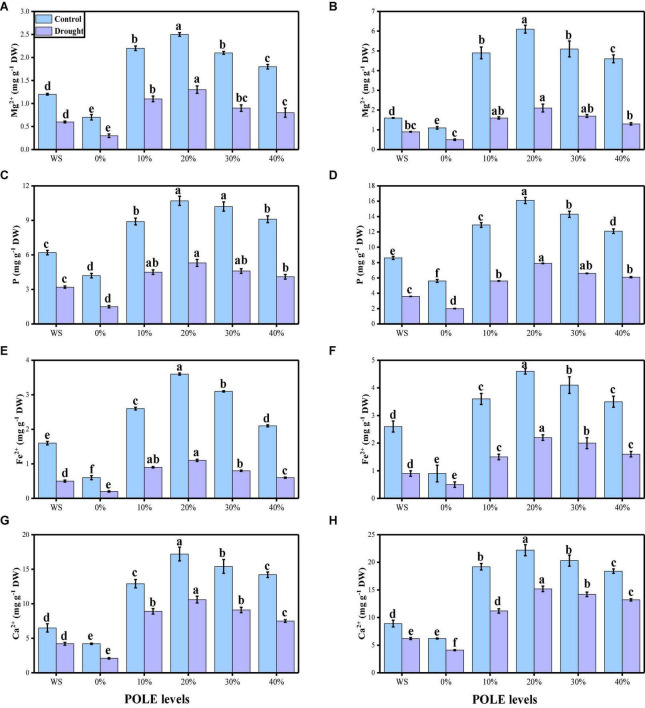
Effect of seed priming with *Plantago ovata* Forsk leaf extract (POLE) on magnesium contents in the roots **(A)**, magnesium contents in the shoots **(B)**, phosphorus contents in the roots **(C)**, phosphorus contents in the shoots **(D)**, iron contents in the roots **(E)**, iron contents in the shoots **(F)**, calcium contents in the roots **(G)**, and calcium contents in the leaves **(H)** under well-watered and drought-stressed environment in wheat. Means sharing similar letter(s) within a column for each parameter do not differ significantly at *P* < 0.05. Data in the figures are means of four repeats (*n* = 4) of just one harvest of wheat plants ± standard deviation (SD). Different lowercase letters on the error bars indicate significant difference between the treatments. Different treatments of POLE used in this study are as follows: WS (Control, without POLE + without water), 0% (0% POLE), 10% (10% POLE), 20% (20% POLE), 30% (30% POLE), and 40% (40% POLE).

The contents of essential minerals such as magnesium (Mg^2+^), phosphorus (P), iron (Fe^2+^), and calcium (Ca^2+^) from the roots and shoots of *T. aestivum* seedlings were also measured in this study. The data regarding the contents of various minerals (Mg^2+^, P, Fe^2+^, and Ca^2+^) in the roots and shoots of wheat seedlings are presented in Figure 8, grown in well-watered and drought-stressed condition under the seed priming with various concentrations of POLE. Compared to the plants grown in the control treatment, the contents of various minerals (Mg^2+^, P, Fe^2+^, and Ca^2+^) in the roots and shoots of wheat seedlings were decreased significantly (*P* < 0.05) in the plants grown in the water-deficient condition. The results show that the contents of various minerals (Mg^2+^, P, Fe^2+^, and Ca^2+^) in the roots and shoots of wheat seedlings were increased with the increasing levels of POLE in control and drought-stressed plants, compared with those plants which were not primed (0% POLE). Moreover, the maximum increased in the contents of various minerals (Mg^2+^, P, Fe^2+^, and Ca^2+^) in the roots and shoots of wheat seedlings were found in the plants which were grown in 20% POLE, while further increase in POLE such as 30 and 40% POLE induce a significant (*P* < 0.05) decrease in the in the contents of various minerals, compared with those plants which were not primed with POLE.

### Relationship between growth, physiology, and nutritional status in various organs of *Triticum aestivum*

**FIGURE 9:**
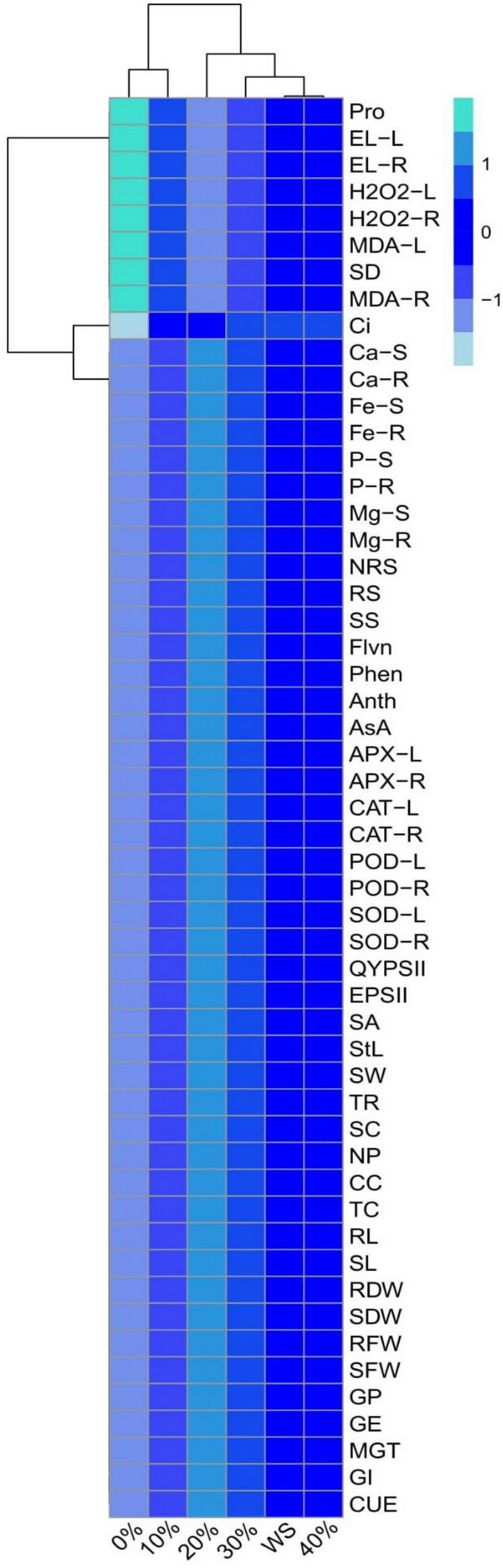
A heatmap between different studied attributes of wheat *grown* under well-watered and drought-stressed environment. Different treatments of *Plantago ovata* Forsk leaf extract (POLE) used in this study are as follows: WS (Control, without POLE + without water), 0% (0% POLE), 10% (10% POLE), 20% (20% POLE), 30% (30% POLE), and 40% (40% POLE). Different abbreviations used are as follows: Pro, proline contents; EL-L, electrolyte leakage in the leaves; EL-R, electrolyte leakage in the roots; H_2_O_2_-L, hydrogen peroxide initiation in the leaves; H_2_O_2_-R, hydrogen peroxide initiation in the roots; MDA-L, malondialdehyde contents in the leaves; SD, stomatal density; MDA-R, malondialdehyde contents in the roots; Ci, intercellular CO_2_; Ca-S, calcium contents in the shoots; Ca-R, calcium contents in the roots; Fe-S, iron contents in the shoots; Fe-R, iron contents in the roots; P-S, phosphorus contents in the shoots; P-R, phosphorus contents in the roots; Mg-S, magnesium contents in the shoots; Mg-R, magnesium contents in the roots; NRS, non-reducing sugars; RS, reducing sugars; SS, soluble sugars; Flvn, flavonoid contents; Phen, phenolic contents; Anth, anthocyanin contents; AsA, ascorbic acid contents; APX-L, ascorbate peroxidase activity in the leaves; APX-R, ascorbate peroxidase activity in the roots; CAT-L, catalase activity in the leaves; CAT-R, catalase activity in the roots; POD-L, peroxidase activity in the leaves; POD-R, peroxidase activity in the roots; SOD-L, superoxidase dismutase activity in the leaves; SOD-R, superoxidase dismutase activity in the roots; QYPSII, quantum yield of PSII; EPSII, efficiency of PSII; SA, stomatal aperture; StL, stomatal length; SW, stomatal width; TR, transpiration rate; SC, stomatal conductance; NP, net photosynthesis; CC, carotenoid contents; TC, total chlorophyll; RL, root length; SL, shoot length; RDW, root dry weight; SDW, shoot dry weight; RFW, root fresh weight; SFW, shoot fresh weight; GP, germination percentage; GE, germination energy; MGT, mean germination time; GI, germination index; and CUE, co-efficient of uniformity of emergence.

A heatmap-histogram analysis was also constructed to explore the relationship between the different growth and ions uptake in various parts of the plants (Figure 9). Heatmap-histogram analysis shows that 0% POLE showed a significant result with various oxidative stress indicators, proline contents while rest of heatmap-histogram is showing non-significant results in all other parameters studied in this study. The color blue in Figure 9 indicates the non-significant results of our study, and the aquamarine color is showing significant results in this figure. This histogram depicted a clear difference between the ion uptake abilities and growth attributes of wheat.

**FIGURE 10:**
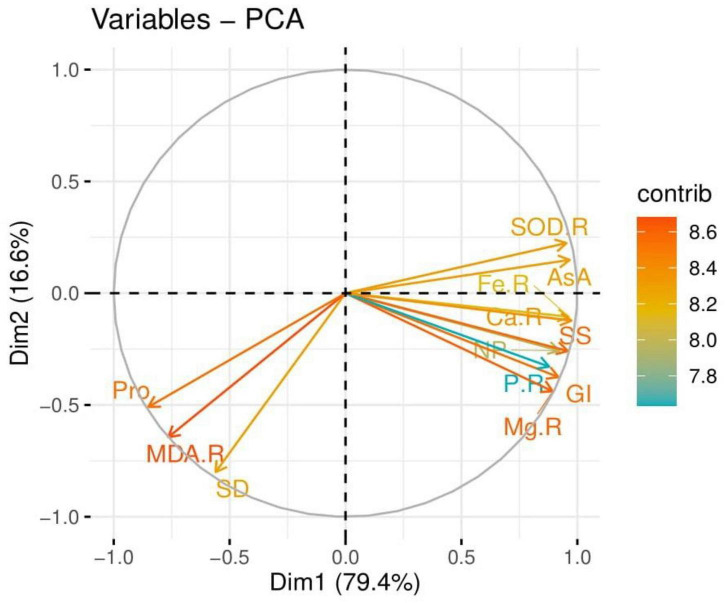
Loading plots of principal component analysis (PCA) on different studied attributes of wheat grown under well-watered and drought-stressed environment with various application levels of *Plantago ovata* Forsk leaf extract (POLE). Different abbreviations used are as follows: Ca-R, calcium contents in the roots; Fe-R, iron contents in the roots; P-R, phosphorus contents in the roots; Mg-R, magnesium contents in the roots; SS, soluble sugar; AsA, ascorbic acid contents; SOD-R, superoxidase dismutase activity in the roots; NP, net photosynthesis; GI, germination index; Pro, proline contents; SD, stomatal density; and MDA-R, malondialdehyde contents in the roots.

The loading plots of principal component analysis (PCA) to check the effect of drought stress with the seed priming with POLE on some selected attributes of wheat seedlings are presented in Figure 10. The loading plots of PCA show that the contents of Ca^2+^, Mg^2+^, P, and Fe^2+^ in the roots and shoots and soluble sugar, ascorbic acid contents, SOD activity in the roots, net photosynthesis, total chlorophyll contents, shoot length, germination index, and shoot fresh weight show a positive correlation with each other while proline contents, stomatal density, and MDA contents in the roots show a negative correlation.

## Discussion

Drought being as a stern environmental setback is associated with the deviation in surface hydrological processes and climate (Ahmad et al., 2017; Hasanuzzaman et al., 2018; Sakya and Prahasto, 2020). Water is one of the most limiting factors that influence many morphological, physiological, biochemical, metabolic, transcriptomic, and proteomic processes, thereby affecting plant survival, development, and crop yields (Fahad et al., 2017; Schmidthoffer et al., 2018; Khan et al., 2019a). In addition, drought induces various changes in morphological and physiological behavior of plants including altered plant water relation, lower growth rate, reduced stem elongation, expansion of leaf, stomatal movement, ion and nutrient imbalance and photosynthesis (Mahajan and Tuteja, 2005; Aslam et al., 2013). Soil drought is a predominant environmental factor that restricts plant growth worldwide and is known to slow plant growth rates and reduce crop yields by inhibiting plants’ carbon assimilation capacity (Bashir et al., 2020; Yaseen et al., 2020; Hussain et al., 2022). In the present study, we have noticed that low water contents in the medium (60% OM) significantly (*P* < 0.05) decrease seed germination, plant growth, and biomass, compared to those plants which were grown in the well-watered condition (Figures 1, 2). Drought indicates the unavailability of water for the plant uptake. Thus, the primary effect of drought stress on the plant is appeared as reduced water content. This reduction of water content resulted in growth and physiological disorder (Khan et al., 2019a). It was also specified that low soil moisture in the soil would suppress root growth, which eventually results in restricting water absorption from the subsoil and changing the plants’ ability to tolerate drought stress (Ahmad et al., 2017; Hasanuzzaman et al., 2018; Taha et al., 2020). Previous studies have documented that water stress hampered growth at seedling stage because of inhibited cell expansion and decrease in carbon acclimatization and partitioning (Nikolaeva et al., 2010; Aslam et al., 2013; Ahmad et al., 2017; Akram et al., 2018; Noman et al., 2018; Khan et al., 2019a).

Although many stress factors suppress plant growth by targeting more than one physiological process, photosynthesis is the most severely affected, as reflected by changes in biomass production that may be due to a lower demand for assimilates (Nikolaeva et al., 2010; Khan et al., 2019b). Under such conditions, photosynthesis is influenced by several mechanisms, including stomatal limitations, the primary drought-stress response that leads to reduced stomatal conductance. When plants close their stomata to minimize water losses, that action also inhibits atmospheric CO_2_ diffusion into the leaf and chloroplasts, thereby decreasing the rate of photosynthesis and limiting carbon assimilation, which then reduces plant growth (Kosar et al., 2015; Saud et al., 2017). We have also noticed in the present study that drought-stressed condition significantly affected photosynthetic pigments, gas exchange parameters, and stomatal properties in wheat seedlings, compared to the plants grown in the well-watered condition (Figures 3, 4). Net photosynthesis is decreased by regulation mechanisms which include decreases in stomatal behavior and photosynthetic pigments when plants are exposed to drought (Liang et al., 2018). Previously, it was observed that normal or controlled water environment showed significantly higher photosynthetic apparatus and stomatal aperture, compared to the plants grown in the water-deficient condition (Khan et al., 2019a; Bashir et al., 2020; Sharma et al., 2020). Similar trend was also observed in *T* wheat seedlings, when exposed to drought stress condition (Mathur et al., 2019; Adrees et al., 2020) due to stomatal closure found due to water shortage as shown by Rivas et al. (2016) in *Vigna unguiculata* species. Moreover, the reduction in transpiration rate might be due to the plants that does not maintain water field capacity which was induced by rate of transpiration (Rivas et al., 2016). When plants undergoes water limited conditions, stomata progressively close, reducing stomatal conductance, resulting in decreased photosynthesis and transpiration as showed by Lotter et al. (2014) in fynbos legume under water-deficient environment.

Oxidation of photosynthetic pigments, membrane lipids, proteins, and nucleic acids are the crucial reasons of plant damage, which occur due to the drought-induced high ROS production leading to the modification of cellular redox status (Saleem et al., 2019a,b, 2020f; Kamal et al., 2022). Low water levels in the soil cause ultra-structural alterations, oxidative stress in plants and increased EL, MDA concentrations, whereas induced alterations in antioxidant enzyme activities are such as SOD, POD, CAT, and APX (Imran et al., 2019; Kamran et al., 2019; Alatawi et al., 2022; Azeem et al., 2022; Naveed et al., 2022). As reported by Taha et al. (2020) antioxidants, such as ascorbic acids and glutathione contents, additional ROS may be scavenged and therefore increased plant tolerance against harsh conditions may be achieved. Furthermore, osmo-protectants also constitute redox active molecules which overcome the production of ROS as well as participate in the ascorbate-glutathione cycle (Rana et al., 2020; Rehman et al., 2020; Imran et al., 2021; Akhtar et al., 2022; Mfarrej et al., 2022). In the present study, the contents of MDA, H_2_O_2_, and EL (%) were increased in the plants which were grown in the drought-stressed environment (Figure 5), compared to those plants which were grown in the well-watered conditions. Plant has strong defense system and increased enzymatic (SOD, POD, CAT, and APX) and non-enzymatic antioxidants (ascorbic acid, anthocyanin, phenolics, and flavonoids) to scavenge ROS production. The increase in the concentration of soluble sugars in drought-stressed plants (Figure 7) shows that improved cell osmotic adjustment would help to maintain higher water content in plant cells and lower electrolyte leakage (Anjum et al., 2017; Hasanuzzaman et al., 2018; Khan et al., 2019b; Afzal et al., 2021; Saleem et al., 2021).

Drought stress reduces plant productivity by inhibiting the uptake of common minerals. Although water uptake and nutrient absorption are independent processes in the roots, the need for water to support nutrient transport and plant growth makes those processes closely related (Waraich et al., 2011; Heile et al., 2021; Mumtaz et al., 2021; Sufyan et al., 2021). Most nutrients are absorbed by plant roots as ions, and water acts as a medium for their movement. Water deficits inhibit this flow of nutrients within the soil, the absorption of those elements, and their uptake by roots (Liang et al., 2018). Therefore, drought-stressed plants will have lower nutrient absorption because less water is available and the diminished power of the roots hinders the uptake process (Waraich et al., 2011). Adequate absorption of minerals is important for the maintenance of plant structural integrity and key physiological processes, and any changes in mineral uptake may negatively affect plant metabolism (Vurukonda et al., 2016; Bashir et al., 2020; Hassan et al., 2021; Javed et al., 2021; Ali et al., 2022a). Compared to the plants grown in the control treatment, the contents of various minerals (Mg^2+^, P, Fe^2+^, and Ca^2+^) in the roots and shoots of wheat seedlings were decreased significantly (*P* < 0.05) in the plants grown in the drought-stressed condition (Figure 8). The plant would take up sufficient quantities of essential nutrients to control plant structure and composition, and many other biological processes of a plant’s life cycle and any decreased in nutrient uptake not only impaired plant metabolism but also decreased plant growth and yield-related attributes (Delshadi et al., 2017; Liang et al., 2018; Ali et al., 2020; Kamran et al., 2020; Yaseen et al., 2020; Rehman et al., 2021).

**FIGURE 11:**
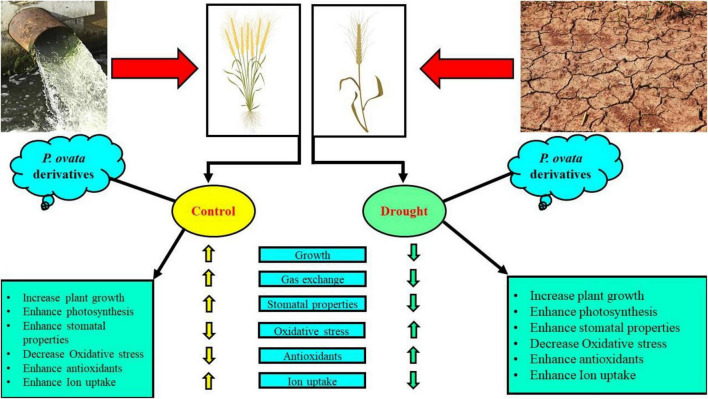
Schematic presentation interpreting the mechanistic role of *Plantago ovata* Forsk leaf extract (POLE) in alleviating the drought stress in wheat. The drought stress inhibited plant growth characteristics and higher ROS concentration was accumulated in the roots and shoots of wheat. In contrast, the application of POLE significantly alleviated the negative impact of drought stress and improved root and shoot growth and alleviated ROS accumulation. POLE addition regulated the antioxidant defense system while reduced the oxidative stress and relative membrane permeability. The current study demonstrated that POLE could relieve drought stress in wheat by reducing oxidative stress at the root surface, and regulating proficient antioxidant coordination in the roots and shoots of wheat.

Recently, priming seeds with different derivatives have become increasingly popular due to the effects of this treatment on plants, which ultimately increase plant growth and biomass in cereal crops (Dawood and El-Awadi, 2015; Abid et al., 2018). *Plantago ovata* leaves extract (POLE) (Psyllium) Forsk is an annual, medicinal (family: Plantaginaceae) and cultivated in the most countries of Asia (most cultivation is found in India) (Patel et al., 2016). Husks of *P. ovata* (also known as isabgol) are rich bioactive compounds and also essential for various primary and secondary metabolites (Talukder et al., 2015; Patel et al., 2016). Under abiotic stress condition such as drought, plants decrease their growth and biomass, due to increasing concentration of ROS which induce oxidative stress in the plants (Khan et al., 2019b; Mohamed et al., 2020). The use of medicinal plant leaf extracts such as *P. ovata* increased enzymatic and non-enzymatic activities in various plant species, which scavenged ROS production and thus ultimately increased growth and biomass due to active defense mechanism (Patel et al., 2016). There is a limited literature available on POLE to ameliorate abiotic stress in the plants, but the results from this study are depicting that this is a promising technique of seed priming and able to increase seed germination (Figure 1), plant growth (Figure 2), photosynthetic measurements (Figure 3), stomatal properties (Figure 4), and ions uptake (Figure 8) and decreasing oxidative stress (Figure 5) by optimizing the activities of various antioxidant compounds (Figures 6, 7) in the different organs of *T. aestivum*. Therefore (Tlili et al., 2019) studied the positive impacts of POLE along with many other medicinal plant extracts and noticed that POLE significantly increased plant growth, antibacterial activities and shrimp toxicity. However, decrease in growth and photosynthesis in severe high contents of 30 and 40% POLE might be due to the excessive amount or toxic amount of POLE to *T. aestivum* seedlings. Although this idea is new, but in another study by Nawaz et al. (2021), they studied the POLE extract to *Zea mays* seedlings under the drought stress and noticed that the drought stress induced a negative impact on plant growth, stomatal properties, gas enhance characteristics, and ion uptake in different organs of *Z. mays* but induced oxidative stress in the plants which were manifested by the increasing the activities of enzymatic and non-enzymatic antioxidant compounds. The use of POLE significantly ameliorates the negative impact of drought by increasing plant growth and biomass, stomatal properties, gas enhanced characteristics, and ion uptake in different parts of plants by decreasing oxidative damage to membrane-bounded organelles and increasing the activities of various antioxidant compounds. The use of POLE decreased the oxidative stress and increased antioxidant capacity, that is, one of the possible factors to enhance the plant growth and photosynthetic pigments in drought-stressed *T. aestivum*. Some previous studies also showed that the use of POLE is able to protect a plant species under low water content in the soil as showed by Rahimi et al. (2010) under different water regimes. These results indicated that POLE addition might alleviate the negative effects of drought stress manipulation on whole-plant growth of the plant. It is demonstrated that POLE addition might play a key role in maintaining plant productivity under different soil water conditions in the arid and semi-arid land. The schematic presentation of *T. aestivum* seedlings grown under the drought stressed with the application of POLE is presented in Figure 11.

## Conclusion

The findings showed that the drought stress significantly (*P* < 0.05) decreased plant growth attributes, photosynthetic efficiency, and nutritional status of the plants, while increased ROS concentration in the plant tissues which induced oxidative damaged in the membranous-bounded organelles. Therefore, the findings also showed that the seed priming with POLE non-significantly (*P* < 0.05) enhanced plant growth and related attributes and ultimately improved plant yield and decreased the oxidative stress indicators by enhancing the activities of various antioxidant compounds. Therefore, long-term field studies should be executed to draw parallels amongst plants/crops nutrients mobility patterns and plant growth in order to gain insights into underlying mechanisms. Hence, based on the present findings, it is recommended that seed priming of field cultivated wheat with 20% POLE should be done to produce crop that is able to tolerate water scarcity.

## Data availability statement

The raw data supporting the conclusions of this article will be made available by the authors, without undue reservation.

## Author contributions

KA, HASA, and AAMA: conceptualization. WAA, SMA, and AAMA: data curation. MHS, DCV, MSA, and RAM: formal analysis. DCV and RAM: funding acquisition. KA, HASA, and AAMA: investigation. WAA and SMA: methodology. MHS, DCV, MSA, and RAM: validation. KA and HASA: writing original draft. KA, HASA, AAMA, WAA, SMA, AAMA, MHS, MSA, DCV and RAM: writing, review, and editing. All authors contributed to the article and approved the submitted version.
