# Supplementary material for: Impact of Plantago ovata Forsk leaf extract on morpho-physio-biochemical attributes, ions uptake and drought resistance of wheat (Triticum aestivum L.) seedlings
Source: Front Plant Sci. 2022 Sep 20;13:999170. doi: 10.3389/fpls.2022.999170 (PMC9531683; doi:10.3389/fpls.2022.999170)
Supplement: Supplementary file 1 [file Data_Sheet_1.docx]

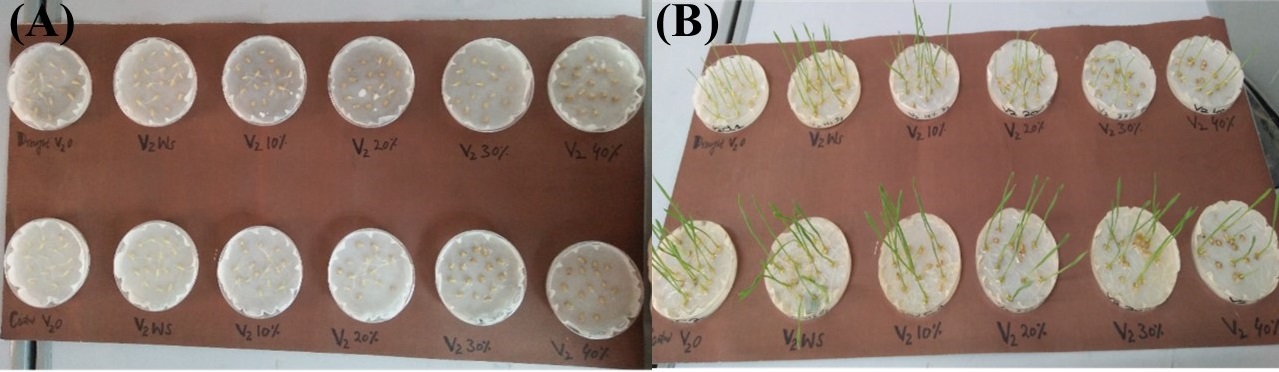


**Figure 1S.** Effect of seed priming with *Plantago ovata* leaf extract on seed germination after 3 days (A) and 10 days (B) under water stressed and non-stressed environment in *T. aestivum*.

**Table 1S.** Chemical composition and concentration of Hoagland’s solution used in the petri dish experiment.

| **Concentration** | **Salts** |
| --- | --- |
| 269.76 | Ca (NO_3_)_2_•4H_2_O |
| 35.15 | KH_2_PO_4_ |
| 48.80 | K_2_SO_4_ |
| 167.68 | CaCl_2_•2H_2_O |
| 324.5 | MgSO_4_•7H_2_O |
| 2.8818 | MnCl_2_•4H_2_O |
| 0.1472 | (NH_4_)6Mo_7_O_24_•4H_2_O |
| 1.8304 | H_3_BO_3_ |
| 0.0704 | ZnSO_4_•7H_2_O |
| 13.33 | Na_2_EDTA |
| 9.96 | FeSO_4_•7H_2_O |
| 0.0629 | CuSO_4_•5H_2_O |
